# Cortical thickness correlated with peripheral inflammatory cytokines in amyotrophic lateral sclerosis

**DOI:** 10.3389/fnins.2024.1514554

**Published:** 2025-01-07

**Authors:** Jing Yang, Wenyi Li, Mei Tian, Lei Zhang, Fengping Du, Xin Li, Qi Liu, Rui Li, Zhenzhong Li, Hui Dong, Yaling Liu

**Affiliations:** ^1^Department of Neurology, The Second Hospital of Hebei Medical University, Shijiazhuang, Hebei, China; ^2^The Key Laboratory of Neurology (Hebei Medical University), Ministry of Education, Shijiazhuang, Hebei, China; ^3^Neurological Laboratory of Hebei Province, Shijiazhuang, Hebei, China; ^4^Department of Emergency, The Second Hospital of Hebei Medical University, Shijiazhuang, Hebei, China

**Keywords:** amyotrophic lateral sclerosis, cortical thickness, disease progression, inflammatory cytokines, MRI

## Abstract

**Introduction:**

Amyotrophic lateral sclerosis (ALS) is a rare, devastating neurodegenerative disease that affects upper and lower motor neurons, resulting in muscle atrophy, spasticity, hyperreflexia, and paralysis. Inflammation plays an important role in the development of ALS, and associated with rapid disease progression. Current observational studies indicate the thinning of cortical thickness in patients with ALS is associated with rapid disease progression and cognitive changes. However, the effects of inflammatory cytokines on cortical thickness in patients with ALS are unclear. Here, we investigated the relationship between inflammatory cytokines and cortical thickness in patients with ALS.

**Methods:**

We evaluated 51 patients with ALS for inflammatory cytokines including interleukin (IL)-4, interferon (IFN)-α, IL-1β, IL-2, IL-5, IL-12, tumor necrosis factor (TNF)-α, IL-6, IL-10, IL-8, IL-17, and IFN-γ and analyzed the correlation between these indicators and the ALS functional rating scale-revised (ALSFRS-R) score or disease progression rate (ΔFS score). Twenty-six patients with ALS and 26 controls were studied using whole-cortex analysis, and *post-hoc* analyses were performed to examine the correlation between brain cortical thickness and ALSFRS-R or ΔFS scores.

**Results:**

IL-4, IFN-α, IL-1β, and IL-2 levels were significantly correlated with ALSFRS-R scores, and the IL-2 level was significantly correlated with ΔFS scores. After controlling for age and sex, the ALS group had thinner cortexes in multiple clusters across the brain than the control group. Further analyses revealed that cortical thickness in the right superior temporal and lingual gyrus regions was inversely correlated with ΔFS scores. There was a significant positive correlation between the clusters in the right lingual cortex and IL-2 level.

**Conclusion:**

These results suggest cortical thickness was reduced in patients with ALS in motor and non-motor cortical areas. Inflammatory factors (especially IL-2) were correlated with cortical thickness, and both were related to the disease progression rate, suggesting IL-2 plays an important role in ALS.

## 1 Introduction

Amyotrophic lateral sclerosis (ALS) is a chronic, adult-onset, progressive, and severe neurodegenerative disease that mainly affects motor neurons (MNs), resulting in muscle atrophy, spasticity, hyperreflexia, and paralysis, with a median survival of 3–5 years (Qureshi et al., 2009). Research has indicated that the pathophysiology of ALS includes oxidative stress, glutamate excitotoxicity, protein homeostasis, defects in RNA processing, impaired axonal transport, and mitochondrial dysfunction (Brown and Al-Chalabi, 2017), but the exact pathogenesis remains unknown. Although Riluzole (Bensimon et al., 1994) and Edaravone (Witzel et al., 2022) have been approved by the U.S. Food and Drug Administration, there is currently no cure or effective treatment for the disease.

Inflammation was previously thought to be the result of protein aggregation in the CNS, but there is growing evidence that immune signaling actually causes the aggregates to form in the earliest stages of the disease process (Zhang et al., 2023). So, inflammation including CNS and peripheral inflammation plays an important role in the development of ALS. Microglia and astroglia have been used as therapeutic targets for patients with ALS (Lall and Baloh, 2017; Dols-Icardo et al., 2020; Migliarini et al., 2021; Yamanaka and Komine, 2018; Izrael et al., 2020). And peripheral immune abnormalities exist in these patients with ALS (Yu et al., 2022; McCombe et al., 2020; Lyon et al., 2019; Beers and Appel, 2019; Yang et al., 2023) and modifying peripheral immune could influence ALS progression (Chiot et al., 2020; Garofalo et al., 2020). Furthermore, a number of studies (Moreau et al., 2005; Sun et al., 2021; Du et al., 2020; Ono et al., 2001) have shown that there is a correlation between serum inflammatory factors and disease severity. In addition, inflammatory cytokines from the periphery can travel to the brain via humoral, neural, and cellular pathways and can affect the function of neurons (Dantzer et al., 2000; Walker et al., 2023).

As the brain imaging technology develops, it is increasingly being used to study ALS (Dadar et al., 2020; Hsueh et al., 2023; Johannes et al., 2013; Li et al., 2023). Cerebral cortex thickness, the distance between the cortical surface and white matter, can be quantified using brain imaging and is a common measure of structural brain changes and a sensitive measure of cortical atrophy (Ambikairajah et al., 2014; Schuster et al., 2014). Several studies (Schuster et al., 2014; Tsermentseli et al., 2012) have found that the thinning of cortical thickness in patients with ALS is associated with rapid disease progression and cognitive changes, and cortical thinning correlated positively with serum neurofilament light chains (Tilsley et al., 2024). However, the association between inflammatory factors and cerebral cortex thickness in these patients is poorly understood.

We hypothesized that a reduction in cortical thickness in patients with ALS is associated with inflammatory factors. In this study, we investigated the correlation between peripheral inflammatory cytokines and cortical thickness in these patients. We also investigated the correlation between peripheral inflammatory cytokines and the rate of ALS progression. Therefore, our results not only further support the role of inflammation in ALS disease progression but also suggest that inflammation participates in the change in cortical thickness, providing new evidence for the use of anti-inflammatory therapy in ALS. Furthermore, the combination of inflammatory factors and cortical thickness may serve as an indicator of ALS disease progression and as a measure of the effectiveness of drugs.

## 2 Materials and methods

### 2.1 Subjects

All patients with ALS in this study were recruited from the Department of Neurology, Second Hospital of Hebei Medical University, between January 2021 and October 2023. Healthy controls were recruited from the Physical Examination Department during the study period. Data including sex, age, height, weight, detailed medical history, and physical, laboratory, and electrophysiological examinations were collected. All patients met the revised diagnostic criteria for ALS (Brooks et al., 2000). The ALS functional rating scale-revised (ALSFRS-R) was applied to assess the severity of the disease (Cedarbaum et al., 1999), and the rate of disease progression was evaluated using the ALSFRS-R score-to-progression rate ratio (ΔFS) according to the following formula: (48-ALSFRS-R score at the time of diagnosis)/(time from onset to diagnosis) (Labra et al., 2016; Westeneng et al., 2018). Patients diagnosed with ALS were included in this study, and the exclusion criteria were: (1) age < 45 years; (2) possible ALS; (3) familial ALS; (4) participants with a ventilator or tracheotomy or combination of these; (5) acute or chronic inflammatory diseases such as acute pneumonia or rheumatoid arthritis; (6) difficulty changing posture in bed; (7) other neurological diseases; (8) claustrophobia; and (9) patient or family members not in agreement. Healthy controls matched by sex and age were recruited, and the exclusion criteria were: (1) age < 45 years; (2) any neurological diseases; (3) refused laboratory tests; (4) any acute or chronic inflammatory diseases; and (5) claustrophobia. The study was approved by the Ethical Committee of the Second Hospital of Hebei Medical University (2023-R248). The enrolled patients underwent a detailed evaluation according to the study protocol shown in Supplementary Figure S1.

### 2.2 Laboratory analyses

Venous blood was drawn from the elbows of the participants in the morning after an overnight fast, and the samples were processed in the laboratory immediately after collection. The cytokine levels were measured and analyzed using a Multiplex Cytokine Assay Kit (XII) (AtomLife, Nanjing, China) according to the manufacturer's instructions. All blood indicators, including IL-5, IL-4, IL-12p70, interferon (IFN)-α, IFN-γ, TNF-α, IL-2, IL-6, IL-1β, IL-10, IL-8, and IL-17, were supplied by the laboratory division of the Second Hospital of Hebei Medical University.

### 2.3 Magnetic resonance imaging acquisition and analysis

All subjects underwent a T1-weighted MRI using a 3-Tesla scanner (Philips Achieva, Best, The Netherlands) with a 8-channel head coil. Three-dimensional T1-weighted images were obtained using a gradient echo sequence with the following parameters: repetition time/echo time, 10.2/4.8 ms; matrix size, 256 × 256 mm; flip angle, 8 degrees; and isotropic voxel size, 1 m3. The T1-weighted data were processed using FreeSurfer 7·2 (http://surfer.nmr.mgh.harvard.edu) with the standard recon-all pipeline. The cortex of the gray matter was parcellated and aligned into 68 distinct regions of interest (ROIs) within the Desikan–Killiany atlas (Desikan et al., 2006). The resulting scans were visually inspected for tissue misclassifications. Manual corrections were performed whenever necessary. The resulting cortical maps were smoothed with a Gaussian kernel of 10 mm full-width half-maximum.

### 2.4 Statistical analysis

Statistical analyses and graphing were performed using GraphPad Prism 9 (GraphPad software) and SPSS26. Continuous variables with normal distributions are presented as means ± standard deviations and with non-normal distributions are presented as medians with interquartile ranges. For normally distributed data, an unpaired *t*-test was used to analyze between-group differences, while Pearson's correlation coefficient was used to analyze correlations. For non-normally distributed data, the Mann–Whitney *U*-test was used for difference analysis, and Spearman's rank correlation coefficient was used for correlation analysis.

We used a general linear model (GLM), controlling for the effects of age and sex on cortical thickness at each vertex across the entire brain, to examine differences in cortical thickness between patients with ALS and healthy controls; for bidirectional effects, a Monte Carlo simulation corrected cluster threshold of *p* < 0.05 was considered to indicate significance (Greve and Fischl, 2018; Hagler et al., 2006). Following identification of statistically significant analyses, *post-hoc* vertex-wise analyses were performed to determine whether the region in the significant findings correlated with inflammatory cytokines. A GLM was created to examine the correlation between ALSFRS-R and ΔFS scores (cortical thickness in a hemisphere-specific vertex-wise analysis), and a Monte Carlo simulation corrected cluster threshold of *p* < 0.05 was considered to indicate significance for bidirectional effects. Statistical significance was set at *p* < 0.05.

## 3 Results

### 3.1 Demographic parameters of healthy controls and patients with ALS

**Figure 1:**
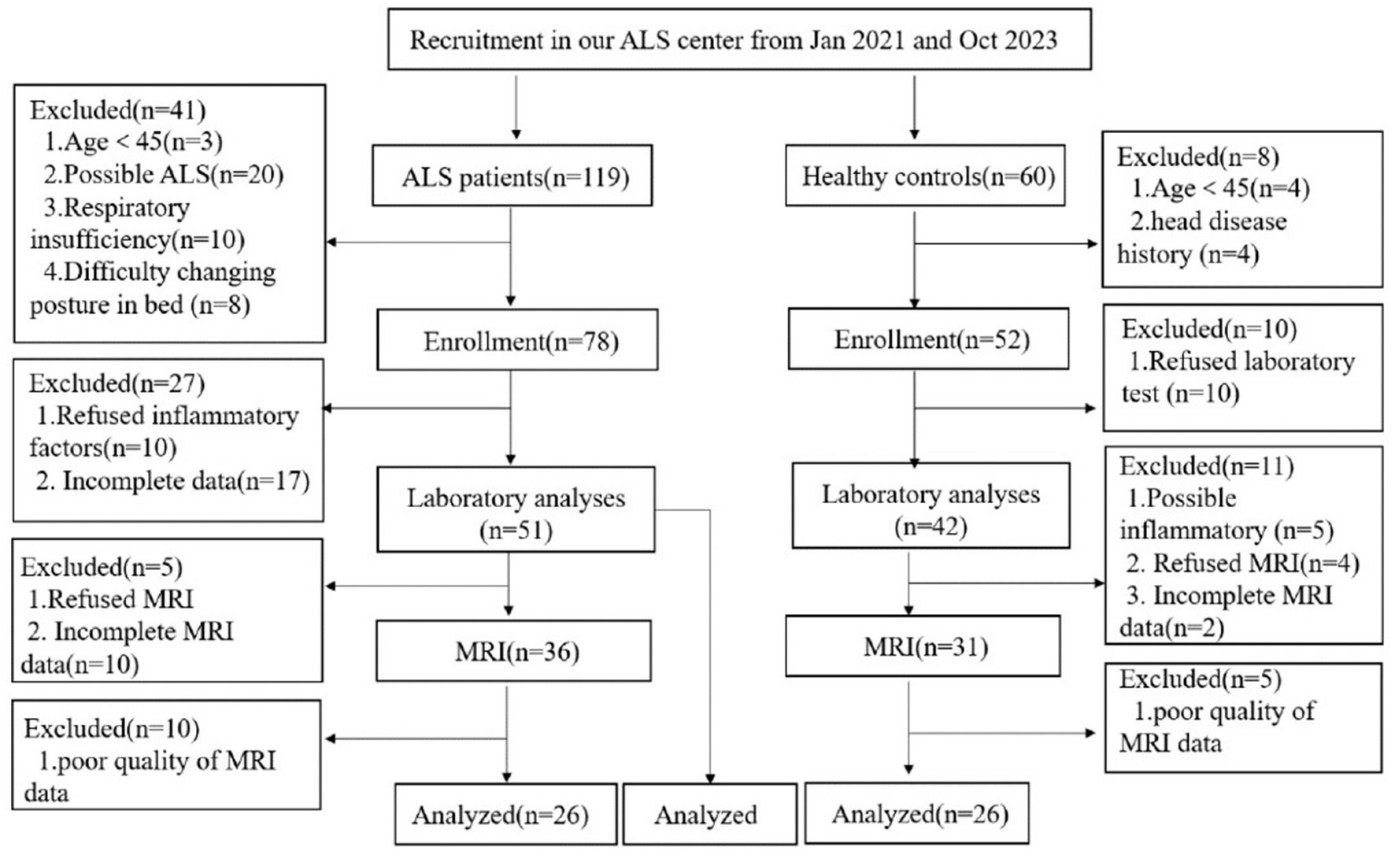
Study flowchart: screening protocol for healthy controls and patients with ALS. ALS, amyotrophic lateral sclerosis; MRI, magnetic resonance imaging.

We screened 119 patients with ALS and 60 healthy controls, of which 51 patients with ALS were included in the correlation analysis between inflammatory factors and the disease progression rate. The healthy control group consisted 13 females and 13 males with a mean age of 56.0 ± 10.8 and a mean education year of 9. The ALS group consisted 24 females and 27 males with a mean age of 59.1 ± 8.7 and a mean education year of 9. Finally, 26 patients with ALS and 26 healthy controls were analyzed (Figure 1). The ALS and healthy controls groups were matched by age, sex, and years of education (Table 1).

**Table 1:** Demographic parameters of healthy controls and ALS patients.

	**Healthy controls (*n* = 26)**	**Patients with ALS (*n* = 51)**	***p*-value**
Age (mean ± SD), y	56.0 ± 10.8	59.1 ± 8.7	0.164
Sex (females/males)	13/13	24/27	0.807
BMI (mean ± SD)	25.04 ± 1.94	23.80 ± 2.78	0.044
**Site of onset**			
Bulbar	NA	16	
Limb	NA	35	
Education years	9, (6, 10)	9 (6, 9)	0.960
Disease duration, m	NA	10 (8, 14)	
ALSFRS-R total score	NA	40 (36, 43)	
ΔFS	NA	0.69 (0.48, 1.13)	
IL-5, pg/ml	NA	2.849 ± 1.608	
IL-4, pg/ml	NA	2.213 ± 1.500	
IL-12, pg/ml	NA	1.954 ± 1.484	
IFN-a, pg/ml	NA	3.262 ± 2.764	
TNF-a, pg/ml	NA	3.376 ± 2.775	
IL-2, pg/ml	NA	3.017 ± 2.757	
IL-6, pg/ml	NA	5.012 ± 3.475	
IL-1β, pg/ml	NA	5.282 ± 6.701	
IL-10, pg/ml	NA	2.553 ± 1.829	
IFN-γ, pg/ml	NA	7.259 ± 6.234	
IL-8, pg/ml	NA	2.308 ± 3.857	
IL-17, pg/ml	NA	5.268 ± 4.820	

Data are presented as means ± SDs or medians (interquartile ranges). ALS, amyotrophic lateral sclerosis; ALSFRS-R, ALS Functional Rating Scale-Revised; BMI, body mass index; ΔFS, [Delta FS ratio (rate of disease progression) = (48-ALSFRS-R score at diagnosis)/time onset to diagnosis].

### 3.2 Correlation between inflammatory factors and disease progression rate

**Figure 2:**
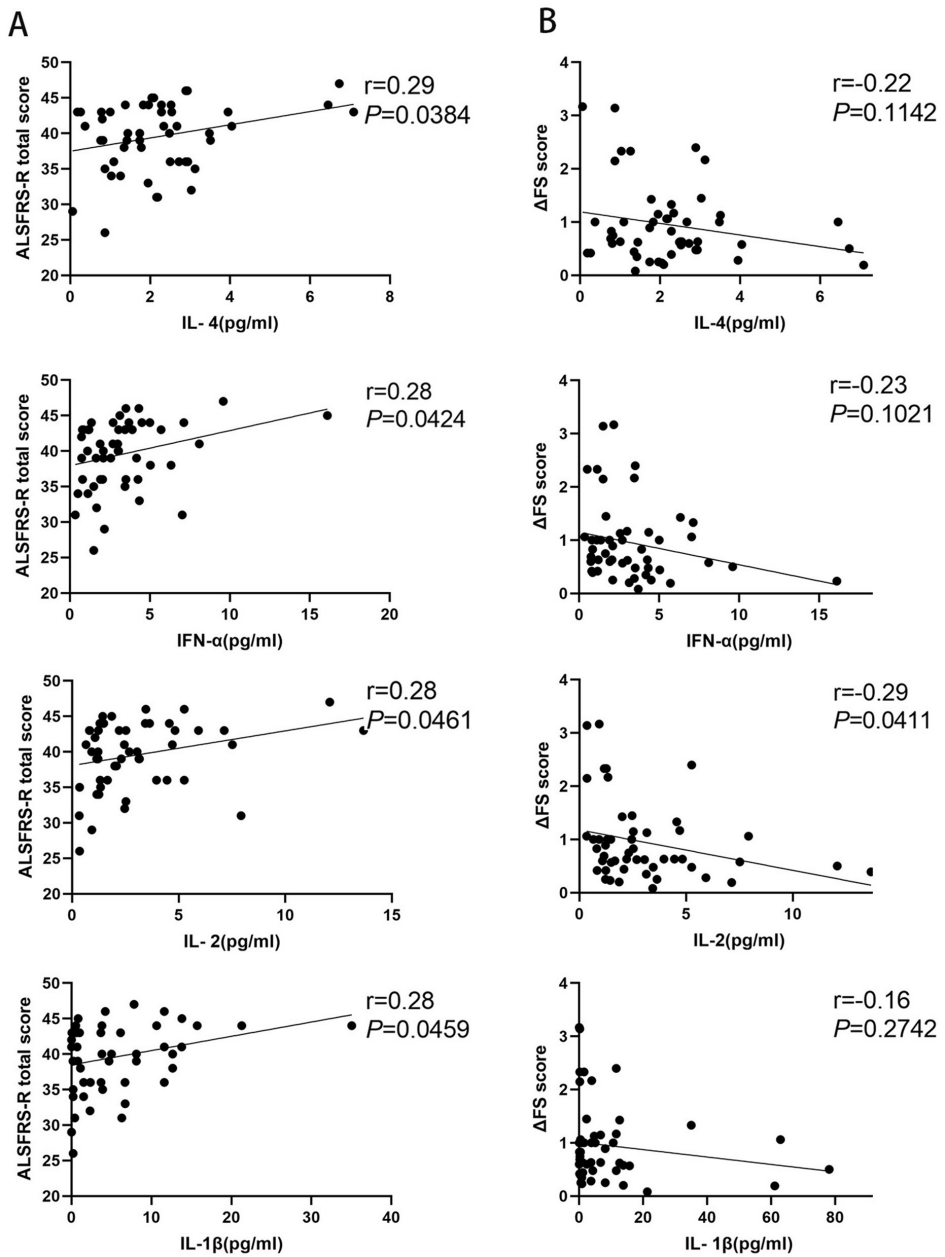
Correlation between inflammatory factors and disease progression rate in patients with amyotrophic lateral sclerosis (ALS). **(A)** The levels of IL-4, IFN-α, IL-1β, and IL-2 in patients with ALS correlated with the amyotrophic lateral sclerosis functional rating scale-revised (ALSFRS-R) scores. **(B)** The levels of IL-4, IFN-α, IL-1β, and IL-2 in patients with ALS correlated with the disease progression rate (ΔFS) scores.

Fifty-one patients with ALS were enrolled to analyze the correlation between inflammatory factors and disease progression rate. We observed that the levels of IL-4, IFN-α, IL-1β, and IL-2 were significantly positively correlated with ALSFRS-R scores (*p* = 0.0384, *p* = 0.0424, *p* = 0.0461, and *p* = 0.0469, respectively), and there was no correlation between the levels of IL-5, IL-12, TNF-α, IL-6, IL-10, IL-8, IL-17, or IFN-γ and ALSFRS-R scores (*p* = 0.2787, *p* = 0.5775, *p* = 0.2753, *p* = 0.7043, *p* = 0.4942, *p* = 0.8496, *p* = 0.1673, and *p* = 0.0788, respectively) (Figure 2A; Supplementary Figure S2A). The IL-2 level was significantly negatively related to the disease progression rate (*p* = 0.0411), but we observed no correlation between IL-4, IFN-α, IL-5, IL-12, TNF-α, IL-6, IL-1β, IL-10, IL-8, IL-17, or IFN-γ and the disease progression rate (*p* = 0.1142, *p* = 0.1021, *p* = 0.8689, *p* = 0.3616, *p* = 0.3588, *p* = 0.7754, *p* = 0.2742, *p* = 0.9703, *p* = 0.8454, *p* = 0.8496, and *p* = 0.6572, respectively) (Figure 2B; Supplementary Figure S2B).

### 3.3 Comparison of cortical thickness between patients with ALS and healthy controls

**Figure 3:**
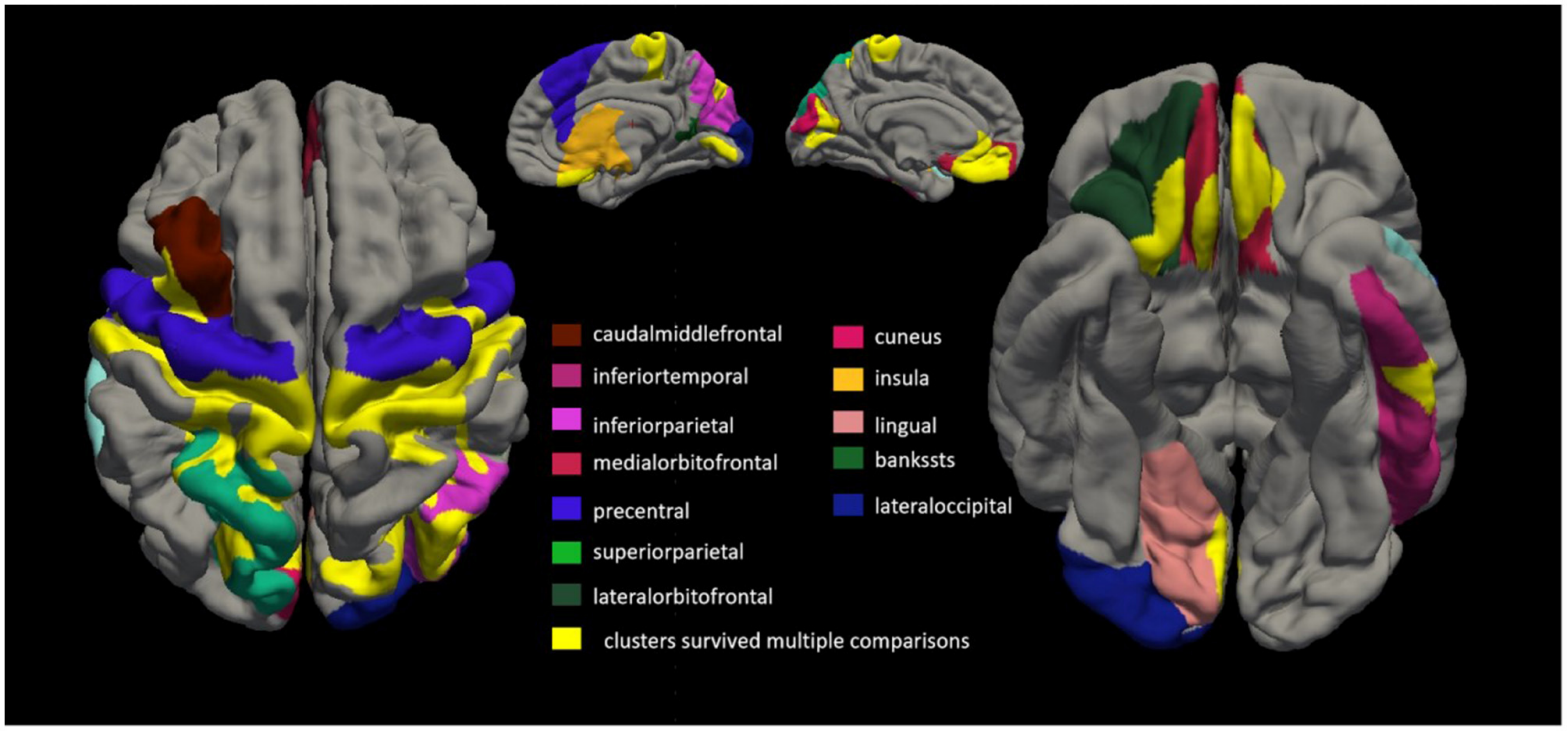
Comparing cortical thickness of patients with amyotrophic lateral sclerosis (ALS) with that of healthy controls. We conducted a vertex-by-vertex general linear model (GLM) analysis, controlling for age and sex. Clusters that survived multiple comparisons using the Monte Carlo simulation (10,000 permutations) are displayed on the cortical surface templates in yellow. In the left hemisphere, these clusters were in the precentral (area, 4,684.52 mm^2^; *p* = 0.01), medial orbitofrontal (area, 1,486.4 mm^2^; *p* = 0.01), cuneus (area, 1,065.32 mm^2^; *p* = 0.01), superior parietal (area 472.37 mm^2^; *p* = 0.01), caudal middle frontal (area, 401.34 mm^2^; *p* = 0.01), inferior temporal (area, 325.6 mm^2^; *p* = 0.01), superior parietal (area, 265.72 mm^2^; *p* = 0.01), and insular (area, 259.39 mm^2^; *p* = 0.01) regions. In the right hemisphere, the clusters were in the precentral (area, 6,290.67 mm^2^; *p* = 0.02), inferior parietal (area, 2,537.78 mm^2^; *p* = 0.02), lateral orbitofrontal (area, 393.17 mm^2^; *p* = 0.02), insular (area, 345.15 mm^2^; *p* = 0.02), lateral occipital (area, 308.15 mm^2^; *p* = 0.01), lingual (area, 291.93 mm^2^; *p* = 0.01), banks of the superior temporal sulcus (bankssts; area, 239.36 mm^2^; *p* = 0.04), and medial orbitofrontal (area, 231 mm^2^; *p* = 0.04) regions.

Twenty-six patients with ALS (55.6 ± 14.4 years of age, 14 men) and 26 matched healthy controls (56.0 ± 10.8 years of age, 13 men) were examined in this study. Compared with the healthy control group, the ALS group had thin cortexes in multiple clusters across the brain after controlling for age and sex. The results are summarized in Table 2 and Figure 3. In the left hemisphere, these clusters were in the precentral (area, 4,684.52 mm^2^; *p* = 0.01), medial orbitofrontal (area, 1,486.4 mm^2^; *p* = 0.01), cuneus (area, 1,065.32 mm^2^; *p* = 0.01), superior parietal (area, 472.37 mm^2^; *p* = 0.01), caudal middle frontal (area, 401.34 mm^2^; *p* = 0.01), inferior temporal (area 325.6 mm^2^; *p* = 0.01), interior parietal (area, 265.72 mm^2^; *p* = 0.01), and insular (area, 259.39 mm^2^; *p* = 0.01) regions. In the right hemisphere, the clusters were in the precentral (area, 6,290.67 mm^2^; *p* = 0.02), inferior parietal (area, 2,537.78 mm^2^; *p* = 0.02), lateral orbitofrontal (area, 393.17 mm^2^; *p* = 0.02), insular (area, 345.15 mm^2^; *p* = 0.02), lateral occipital (area, 308.15 mm^2^; *p* = 0.01), lingual (area, 291.93 mm^2^; *p* = 0.01), banks of the superior temporal sulcus (bankssts; area, 239.36 mm^2^; *p* = 0.04), and medial orbitofrontal (area, 231 mm^2^; *p* = 0.04) regions.

**Table 2:** Significant clusters exhibiting reduced cortical thickness in patients with ALS compared with healthy controls.

**Brain regions**	**Cluster size (mm^2^)**	**Peak coordinates in MNI space (mm) (*****x***, ***y***, ***z*****)**			***p*-value**
LT precentral	4,684.52	−38.5	−16.6	31.3	0.01
LT medialorbitofrontal	1,486.4	−5.2	25.1	−21.4	0.01
LT cuneus	1,065.32	−4.4	−69.3	18.7	0.01
LT superiorparietal	472.37	−23.5	−71.1	27.5	0.01
LT caudalmiddlefrontal	401.34	−38.6	8.2	47.7	0.01
LT inferiortemporal	325.6	−53.1	−23.8	−20.6	0.01
LT superiorparietal	265.72	−13.1	−50.1	58.1	0.01
LT insula	259.39	−36.6	−6.2	0.2	0.01
RT precentral	6,290.67	40	−12.6	29.8	0.01
RT inferiorparietal	2,537.78	45.1	−58.5	35.4	0.01
RT lateralorbitofrontal	393.17	13.4	22.6	−21.5	0.01
RT insula	345.15	37.2	−5.1	−0.3	0.01
RT lateraloccipital	308.15	40.7	−69.6	0.9	0.01
RT lingual	291.93	4.1	−78.2	2.7	0.01
RT inferiorparietal	274.33	44.7	−54.3	15.2	0.01
RT bankssts	239.36	45.7	−39.9	13.4	0.04
RT medialorbitofrontal	231	6.6	18.5	−19	0.04

*P*-values represent whole-brain corrected values based on cluster-wise correction; MNI, Montreal Neurological Institute; RT, right; LT, left.

### 3.4 Analyses of correlation between cortical thickness and disease progression rate

**Figure 4:**
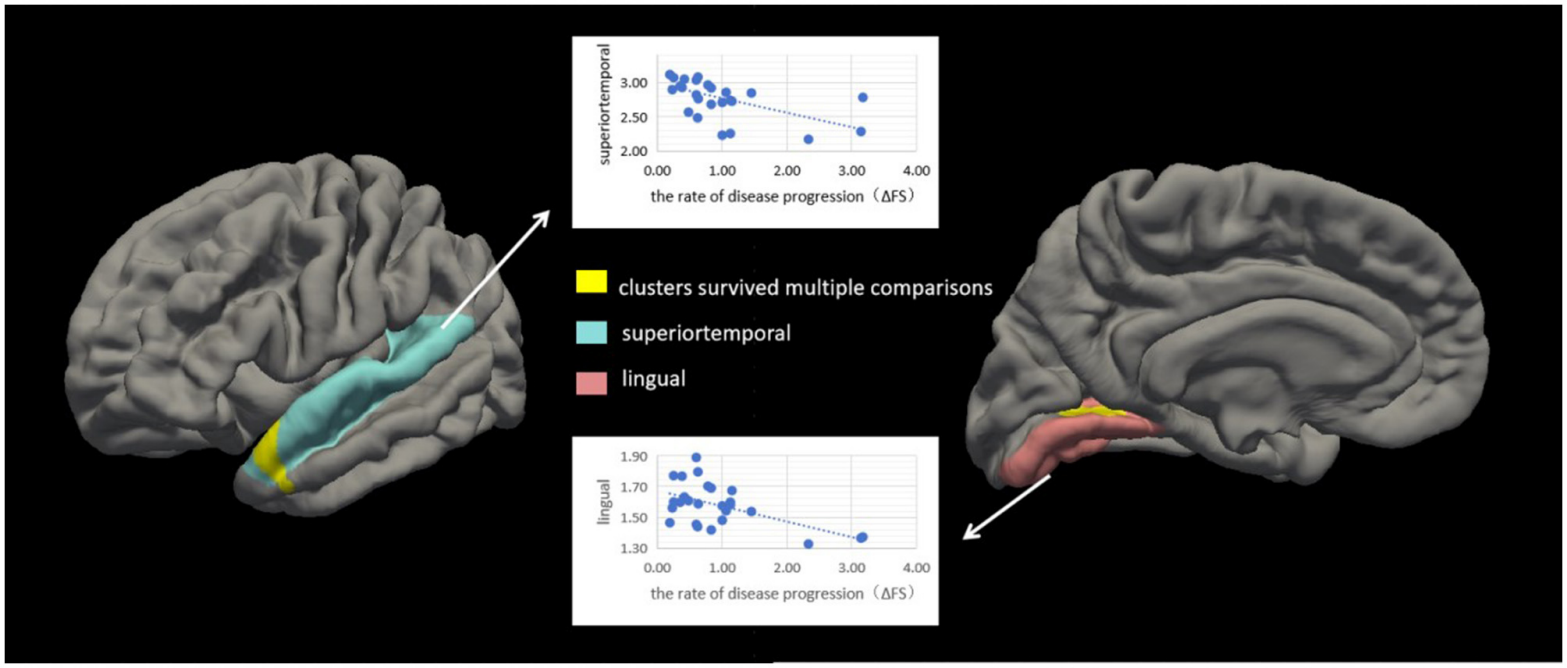
Correlation between cortical thickness and ΔFS scores in patients with amyotrophic lateral sclerosis (ALS). Significant clusters (yellow) exhibiting cortical thickness in the left hemisphere were negatively correlated with ΔFS scores in patients with ALS. In the left hemisphere, these clusters were in the superior temporal (area, 333.61 mm^2^; *p* = 0.01) and lingual (area, 214.73 mm^2^; *p* = 0.04) regions. There were no other significant associations between ΔFS scores and cortical thickness in the right hemisphere.

We conducted correlation analyses to examine the association of cortical thickness with ΔFS. Significant clusters exhibited an inverse correlation between cortical thickness and ΔFS scores (Table 3; Figure 4). In the right hemisphere, these clusters were in the superior temporal (area, 333.61 mm^2^; *p* = 0.01) and lingual (area, 214.73 mm^2^; *p* = 0.04) regions. There were no other significant associations between ΔFS scores and cortical thickness in the left hemisphere and no significant associations between ALSFRS-R scores and cortical thickness across the whole brain.

**Table 3:** Significant clusters exhibiting cortical thickness correlates with ΔFS in patients with ALS.

**Brain regions**	**Cluster size (mm^2^)**	**Peak coordinates in MNI space (mm) (*****x***, ***y***, ***z*****)**			***P*-value**
RT superiortemporal	333.61	−47	3.3	−16.5	0.01
RT lingual	214.73	−16.5	−67.6	4.6	0.04

*P*-values represent whole-brain corrected values based on cluster-wise correction. MNI, Montreal Neurological Institute. RT, right; ΔFS, [Delta FS ratio (rate of disease progression) = (48-ALSFRS-R score at diagnosis)/time of onset to diagnosis].

### 3.5 Analyses of correlation between cortical thickness and inflammatory cytokine levels

Accumulating evidence suggests that inflammation, including that in the CNS and peripheral immune system, contributes to the progression of ALS in both humans and mouse models (McCombe et al., 2020; Beers and Appel, 2019; Lu et al., 2016; Alexianu et al., 2001). This raises the question of whether inflammatory cytokine levels are related to cortical thickness.

**Figure 5:**
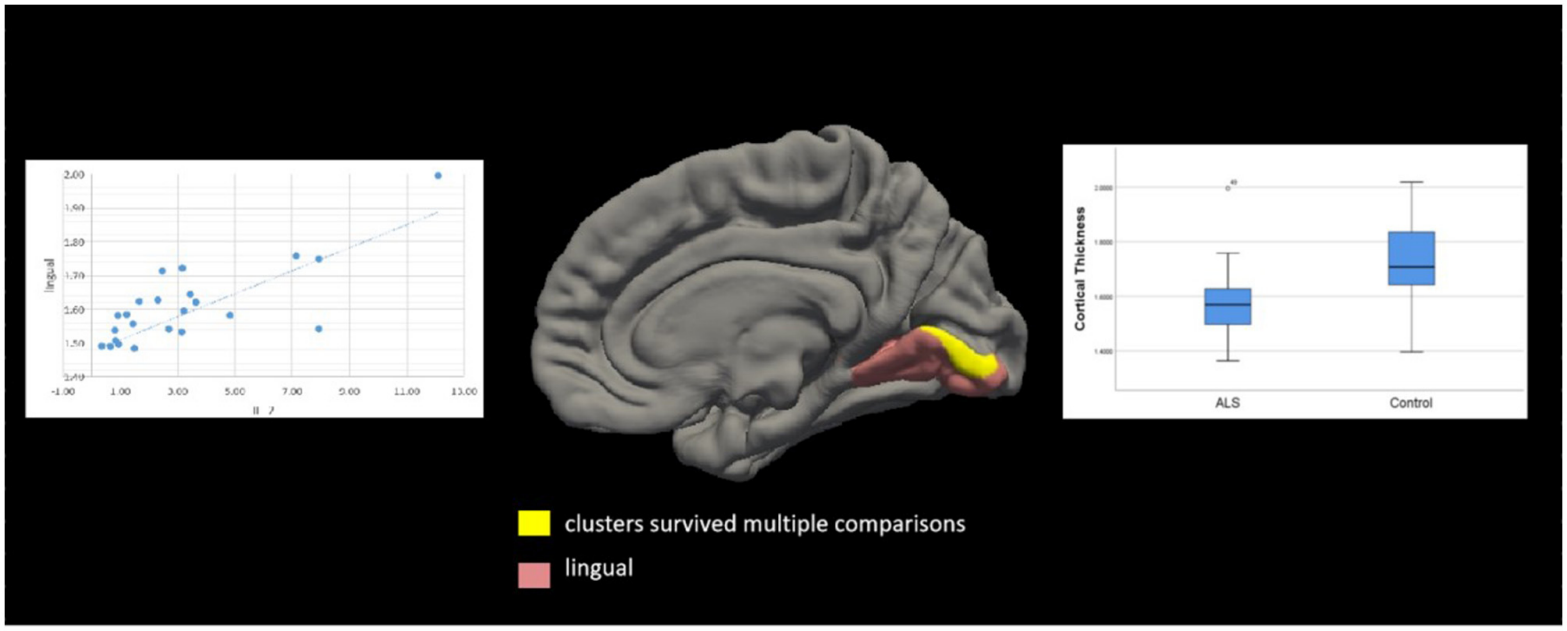
Correlation between cortical thickness and IL-2 level in patients with amyotrophic lateral sclerosis (ALS). Significant clusters (yellow) exhibiting cortical thickness in the right hemisphere were positively correlated with IL-2 levels in patients with ALS after controlling for age and sex. These clusters were in the left lingual region (area, 200.28 mm^2^; *p* = 0.03). Box and whisker plots show the median, quartiles, and range of the significant clusters for lingual cortical thickness in 26 healthy controls and 26 patients with ALS. There were no other significant associations between the IL-2 level and cortical thickness in the left hemisphere.

Based on the whole-cortex analysis results, regions were selected from the Desikan–Killiany atlas to serve as ROIs for the constrained exploratory *post-hoc* analyses. The purpose of this exploratory *post-hoc* ROI analysis was to identify significant associations between the ROIs (thinner cortex in patients with ALS) and IL-2 levels to test the hypothesis that IL-2 is protective in patients with ALS. Based on the whole-cortex analysis results (Table 2), the following ROIs were selected: the left and right precentral, left and right insular, left and right medial orbitofrontal, left cuneus, left superior parietal, left caudal middle frontal, left inferior temporal, right inferior parietal, right lateral orbitofrontal, right lateral occipital, right lingual, and right banksst regions. A positive two-tailed cluster of 200.28 mm^2^ (*p* = 0.05) (Table 4; Figure 5) was found for the cortical thickness of the ROIs in the right hemisphere and IL-2 level after correction for multiple comparisons and controlling for age and sex.

**Table 4:** Significant clusters exhibiting cortical thickness correlates with IL-2 in patients with ALS.

**Brain regions**	**Cluster size (mm^2^)**	**Peak coordinates in MNI space (mm) (*****x***, ***y***, ***z*****)**			***p-*value**
RT lingual	200.28	5.0	−81.1	2.4	0.03

*P*-values represent ROI corrected values based on cluster-wise correction. MNI, Montreal Neurological Institute. LT, left.

## 4 Discussion

Increasing evidence suggests that patients with ALS have chronic inflammation (Moreau et al., 2005; Sun et al., 2021; Pronto-Laborinho et al., 2019; Lunetta et al., 2017). It has previously been shown that these patients have elevated levels of certain cytokines, such as IL-2, IL-6, IL-10, IL-1β, IL-8, TNF-α, and IFN-γ, compared with controls (Lu et al., 2016; Tortelli et al., 2020; Hu et al., 2017). In this study, the healthy controls included were recruited from the Physical Examination Department during the study period, and in screening the healthy controls we excluded people with other neurological diseases and any acute or chronic inflammatory diseases, so we considered the cytokine levels in the peripheral blood of the controls to be very low. And we focused on the correlation between the cytokine levels and ALS disease and did not compare the cytokine levels between ALS patients and healthy controls, so we did not test the cytokine levels in the peripheral blood of healthy controls. Several studies (Moreau et al., 2005; Sun et al., 2021; Du et al., 2020; Ono et al., 2001) have also shown that elevated serum IL-6 was associated with disease severity in patients with ALS. IL-2 and IL-6 levels have also been proposed as biomarkers for ALS severity (Sun et al., 2021). In contrast to the results of previous studies, in addition to IL-2 being associated with disease severity, we found that IL-4, IFN-γ, and IL-1β were significantly correlated with disease severity in these patients. These results further support the hypothesis that patients with ALS exhibit inflammatory responses. Our analyses revealed that the IL-2 level was significantly and negatively correlated with the rate of disease progression in ALS. However, although the significant *p*-values in our results indicate that there is a correlation between peripheral inflammatory cytokines and the disease progression rate, the lower r indicate that the correlation between the two is weak, which may be due to the small number of samples we collected or the different detection methods. Unfortunately, we did not perform continuous monitoring of inflammatory factors in the peripheral blood of patients with ALS, so the result we obtained was only a possible correlation between inflammatory factors and disease severity or the disease progression rate. Our results provide further support for the involvement of peripheral immune cells in the pathogenesis of ALS. Previous studies have shown that Treg levels are reduced in patients with ALS and are correlated with disease severity and that decreased Treg levels may be a predictor of disease progression and poor survival (Beers et al., 2017). IL-2 is a cytokine required for the production, activation, and survival of Tregs (Pol et al., 2020), suggesting it may be a potential therapeutic drug. Previous studies have shown that low-dose IL-2 is well-tolerated and immunologically effective in patients with ALS (Camu et al., 2020), and Low-dose IL-2 promotes regulatory T-cell expansion and enhances the anti-inflammatory effects of regulatory T cells (Giovannelli et al., 2021). These findings support the possible role of inflammation in the mechanism and progression of the disease and confirm that inflammatory cytokines are elevated in patients with ALS. However, larger sample sizes and additional mechanistic studies are required to investigate the exact role of inflammation in ALS.

Consistent with the results of previous studies (Roccatagliata et al., 2009; Machts et al., 2018; Loewe et al., 2017; Kocar et al., 2021), we found that, compared with the healthy control group, the ALS group had thin cortexes in multiple clusters across the brain after controlling for age and sex; this included the precentral, medial orbitofrontal, cuneus, superior parietal, caudal middle frontal, inferior temporal, and insular regions in the left hemisphere and the precentral, inferior parietal, lateral orbitofrontal, insular, lateral occipital, lingual, bankssts, and medial orbitofrontal regions in the right hemisphere, suggesting that patients with ALS may have structural cortical changes and further substantiating extra motor involvement in ALS (Johannes et al., 2013). We found that cortical thickness in the superior temporal and lingual gyrus regions in the right hemisphere was inversely correlated with ΔFS scores in patients with ALS. However, there were no other significant associations between ΔFS scores and cortical thickness in the left hemisphere and no significant associations between ALSFRS-R scores and cortical thickness across the whole brain. It remains unclear whether changes in the cortical structure are a primary phenomenon or occur secondary to ALS. Many other studies showed that cortical thinning in the bilateral precentral gyrus, right precuneus, and right frontal and temporal lobes, and which was associated with cognitive status (Schuster et al., 2014). In this study, we not only observed the thinning of several cerebral cortical thicknesses in patients with ALS, but also explored the relationship between this altered cortical thickness and inflammatory factors.

We chose a thinner cortex for exploratory *post-hoc* ROI analysis and found that a positive cluster (area, 200.28 mm^2^; *p* = 0.05) in the right lingual cortex was correlated with the IL-2 level. We found that significant clusters located in the right superior temporal (area, 333.61 mm^2^; *p* = 0.01) and right lingual (area, 214.73 mm^2^; *p* = 0.04) regions were inversely correlated with ΔFS scores in patients with ALS. Atrophy in the lingual region in patients with ALS should attract our attention and our results suggest that the administration of IL-2 to patients with ALS may not only modulate Tregs but also may improve the cortical thickness. In subsequent studies, we will add scales and test indicators related to speech screening to assess patients' speech and language functions.

This study has some limitations. First, the sample size was relatively small, which may have led to the lower r. This means that our data may not be fully representative, which may have affected the generalizability of the results. Second, this study did not provide a detailed analysis of the relationship between peripheral inflammatory cytokines and cortical thickness, nor did it rule out the influence of possible confounding factors or explore the potential mechanisms. Third, there is a lack of information regarding semantic deficits in patients with ALS, resulting in the inability to analyse the lingual gyrus using MRI. Therefore, a larger sample size is required to reliably determine the correlation between peripheral inflammatory cytokine levels and cortical thickness in patients with ALS. In subsequent experiments, we will follow patients longitudinally to assess the long-term effects of inflammatory cytokines on the prognosis of these patients, as well as changes in inflammatory cytokines over time. We will also perform cellular and animal experiments to explore the possible mechanisms and identify potential targets.

Alterations in the immune system occur in ALS and may contribute to its pathological features. Our data demonstrated a relationship between peripheral inflammatory cytokines and cortical thickness in patients with ALS, further emphasizing the contribution of peripheral inflammatory cytokines to physiological processes. The IL-2 level was significantly and negatively correlated with the rate of disease progression in patients with ALS and positively correlated with cortical thickness in the right lingual region, suggesting that IL-2 is protective in these patients and that IL-2 biologics may be used in the treatment of ALS. These findings provide additional evidence for the involvement of peripheral immune cells in ALS and provide new insights for peripheral immunotherapy in ALS, which we will further verify in a follow-up study.

## Data Availability

The raw data supporting the conclusions of this article will be made available by the authors, without undue reservation.
