# Supplementary material for: Cortical thickness correlated with peripheral inflammatory cytokines in amyotrophic lateral sclerosis
Source: Front Neurosci. 2025 Jan 7;18:1514554. doi: 10.3389/fnins.2024.1514554 (PMC11747150; doi:10.3389/fnins.2024.1514554)
Supplement: Supplementary file 1 [file Data_Sheet_1.pdf]

Supplementary Material

1.Supplementary Figures

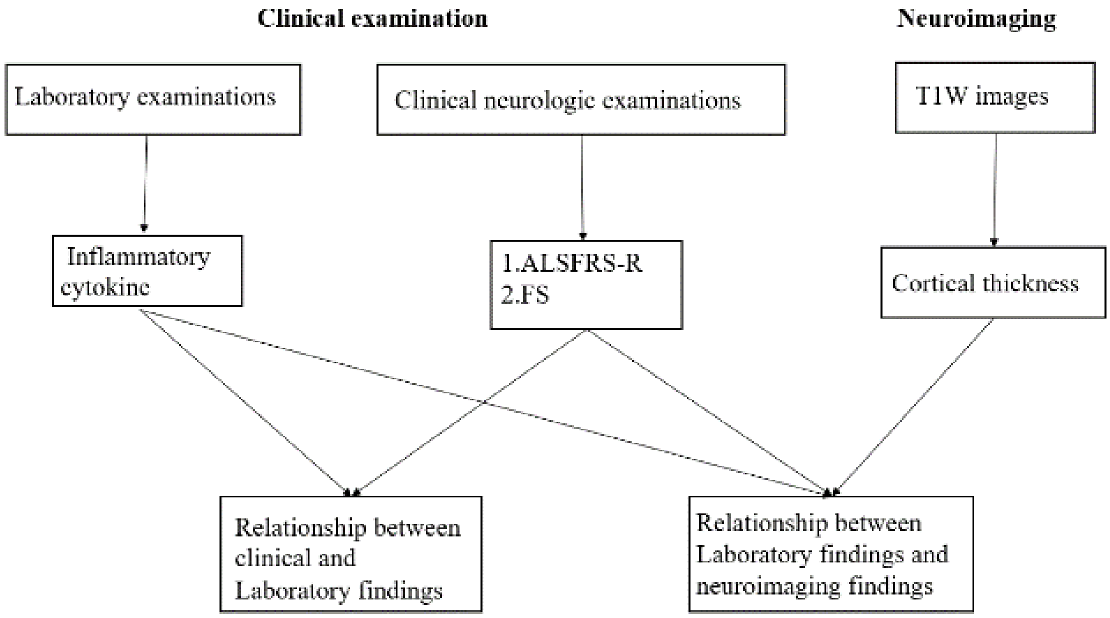

Supplementary Figure 1. Study design to examine the association between neuroimaging parameters and clinical parameters in patients with amyotrophic lateral sclerosis (ALS). Clinical examinations included neurological examinations consisting of the amyotrophic lateral sclerosis functional rating scale-revised (ALSFRS-R) and disease progression rate (ΔFS) scores and laboratory examinations to measure inflammatory cytokine levels. The neuroimaging parameters are presented as cortical thickness.

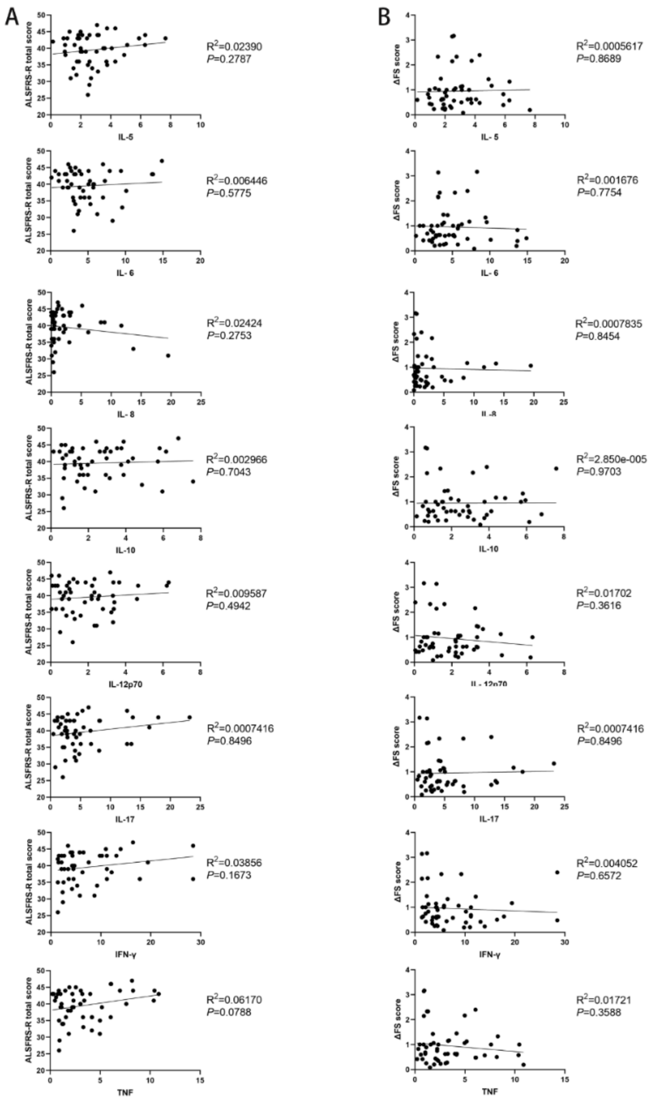

Supplementary Figure 2. Correlation between inflammatory factors and disease progression rate in patients with amyotrophic lateral sclerosis (ALS). (A) The levels of IL-5, IL-12, TNF-α, IL-6, IL-10, IL-8, IL-17, and IFN-γ in patients with ALS correlate with the ALS functional rating scale-revised (ALSFRS-R) scores. (B) The levels of IL-5, IL-12, TNF-α, IL-6, IL-1β, IL-10, IL-8, IL-17, and IFN-γ in patients with ALS correlate with the disease progression rate (ΔFS) scores.
